# Anticancer Potential of Atractylenolides I–III: Efficacy, Mechanisms, Pharmacokinetics, and Safety

**DOI:** 10.3390/molecules31020246

**Published:** 2026-01-11

**Authors:** Lujia Zhang, Jinjian Lu, Mengning Zhang, Yingying Dong, Yutao Luo, Tiantian Lei, Zhujun Bian, Xiaofeng Yuan, Hong Zhao

**Affiliations:** 1 The First School of Clinical Medicine, Zhejiang Chinese Medical University, Hangzhou 310053, China; zhanglujia@zcmu.edu.cn (L.Z.);; 2 State Key Laboratory of Quality Research in Chinese Medicine, Institute of Chinese Medical Sciences, University of Macau, Macao 999078, China; 3 School of Life Sciences, Zhejiang Chinese Medical University, Hangzhou 310053, China

**Keywords:** atractylenolides (I, II, and III), cancer, efficacy, mechanism, pharmacokinetics, safety

## Abstract

Atractylenolides (ATs; mainly AT-I, II, and III), as one of the primary active components of the traditional Chinese medicine *Atractylodes macrocephala*, have demonstrated significant antitumorigenic effects against various cancer cells in both in vitro and in vivo studies. This review aims to systematically review the antitumorigenic effects, mechanisms, pharmacokinetics, and safety profile of ATs, aiming to contribute to clinical research and applications. To achieve this, a systematic literature search was conducted across multiple databases, and findings were synthesized narratively to provide a comprehensive overview of the current evidence. This review comprehensively discusses the antitumorigenic effects and mechanisms of ATs, including arresting tumor cell cycle progression, inducing programmed cell death (apoptosis, autophagy, and ferroptosis), inhibiting tumor angiogenesis, suppressing tumor migration and invasion, modulating the tumor immune microenvironment, and enhancing the efficacy of combination therapies. Additionally, their pharmacokinetic properties and safety profile are summarized, with a focus on their research and application prospects. ATs appear to be safe and reliable candidate anticancer agents in preclinical models, exhibiting potent antitumor efficacy both as monotherapy and in combination regimens. Preliminary clinical data from a small pilot study also indicated no signs of toxicity, but more extensive trials are needed to confirm their safety profile in humans. Further studies on their mechanisms are warranted to facilitate their development into clinically effective antitumor agents.

## 1. Introduction

**Figure 1:**
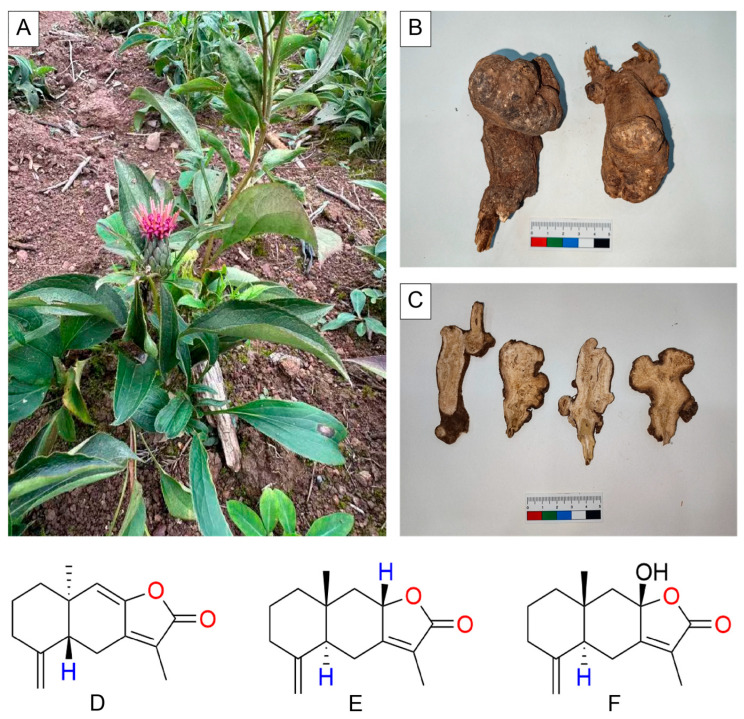
The above-ground portion (**A**), medicinal portion (**B**), and commercial herbal pieces (**C**) of *A. macrocephala*. Molecular formulae of the primary Atractylenolide compounds: Atractylenolide-I (**D**), Atractylenolide-II (**E**), and Atractylenolide-III (**F**).

*Atractylodes macrocephala* (*A. macrocephala*, known as Baizhu in Chinese; Figure 1), the dried rhizome of an Asteraceae species, is a traditional herbal medicine indigenous to East Asia, particularly in eastern and central China, with a well-documented history of clinical applications [1]. According to the Traditional Chinese Medicine (TCM) theory, *A. macrocephala* exerts multiple therapeutic effects, including spleen-strengthening and qi-replenishing effects, dampness-eliminating and diuresis-promoting actions, sweat-inhibiting capacity, and miscarriage-preventing function. Contemporary pharmacological investigations have revealed that it possesses diverse biological activities, including improving gastrointestinal function [2] and antitumorigenic [3], anti-inflammatory [4], anti-aging [5], antioxidant [6], anti-osteoporotic [7], antimicrobial [8], neuroprotective [9], immunoregulatory [10], and energy metabolism-modulating effects [2]. These pharmacological properties contribute to its therapeutic efficacy against various pathological conditions, including malignancies [11], diabetes mellitus [12], hepatitis [13,14], gastritis [15], colitis [10,16], irritable bowel syndrome [17], and constipation [18]. Phytochemical analyses have identified numerous bioactive constituents in *A. macrocephala*, comprising sesquiterpenoids, triterpenoids, polyacetylenes, phenylpropanoids, coumarins, flavonoids, flavonoid glycosides, steroids, benzoquinones, and polysaccharides [19]. The observed pharmacological activities are presumably attributable to these bioactive compounds.

Atractylenolides (ATs), as a class of sesquiterpene lactones, are one of the core pharmacological components of *A. macrocephala* and principally consist of Atractylenolide I, II, and III (Figure 1) [20,21]. Notably, they exert anti-inflammatory [22], anticancer [23,24,25,26], antiviral [27], antiplatelet [28], anti-radiation [29], organoprotective [30,31], and glucose- and lipid-regulating effects [32,33], and have demonstrated therapeutic potential in various diseases including malignancies [34], fatty liver [33], acute liver injury [35], osteoarthritis [36], enteritis [37], allergies [38], gastric ulcers [39], cardiac developmental abnormalities [32], stroke [40], depression [41], spinal cord injury [42], hyperaldosteronism [43], and silicosis [44].

As a result, ATs have been incorporated in numerous classical TCM formulations and represent one of the commonly used clinical components [45,46,47,48]. Regarding their antitumor effects, ATs have demonstrated significant anticancer efficacy across various in vitro cell line models, such as bladder cancer (T-24, 5637), breast cancer (MDA-MB-231), leukemia (HL-60), and lung cancer (A549) cells [23,24,25,26], while also exhibiting favorable anticancer activity in animal studies, including bladder cancer [23] and melanoma [49]. However, the precise mechanisms underlying their antitumor actions remain elusive. Although previous reviews have summarized the general pharmacology of *A. macrocephala* or ATs, a dedicated, comprehensive synthesis focusing specifically on their anticancer potential—integrating efficacy, mechanisms, pharmacokinetics, and safety—is lacking. This review aims to fill this gap, providing an updated and critical evaluation to inform future research and potential translational development.

To ensure comprehensive coverage of relevant studies, a systematic literature search was conducted up to June 2025 using the PubMed, Web of Science, and CNKI databases. The search employed a combination of keywords including “atractylenolide I/II/III”, “AT-I”, “AT-II”, “AT-III”, “cancer”, “antitumor”, “mechanism”, “pharmacokinetics”, and “safety”. Both in vitro and in vivo studies published in English were considered. The inclusion criteria focused on original research articles investigating the anticancer effects, mechanisms, pharmacokinetics, or toxicity of AT-I, AT-II, or AT-III. Review articles, non-cancer studies, and duplicates were excluded. This search and selection framework aimed to provide a structured basis for this integrative review.

## 2. Antitumor Efficacy

### 2.1. Efficacy When Used Alone

ATs have demonstrated anticancer activity both in vivo and in vitro. Indeed, they can inhibit the proliferative capacity of various cancer cells, including breast cancer [24,50,51], colorectal cancer (CRC) [52,53,54,55,56,57], Breast cancer [23], kidney cancer [58], endometrial cancer [59], melanoma [60], lung cancer [26], leukemia [25], ovarian cancer [61], prostate cancer [62], hepatocellular carcinoma (HCC) [63], and gastric cancer [64]. Their antitumor activity performance is affected by cancer type, cell line, duration of exposure, and assay method, as listed in Table 1, with therapeutic effects generally improving with higher intervention doses and durations. Among them, AT-I shows the broadest anticancer effects, demonstrating particular sensitivity in bladder cancer T-24 and 5637 cells [23], breast cancer MDA-MB-231 cells [24], leukemia HL-60 cells [25], and lung cancer A549 cells [26]. Conversely, AT-II appears to confer more obvious therapeutic effects in melanoma [65] and prostate cancer [62] studies. Reports on AT-III in this regard are relatively scarce, the existing evidence indicates that AT-III exerts more limited anti-proliferative effects in vitro compared to AT-I and AT-II, with reported IC_50_ values often exceeding 100 μM in various cancer cell lines [66,67]. Relatively significant activity was recorded in specific models. For example, in human CRC HCT-116 cells, AT-III inhibited proliferation with an IC_50_ of 111.47 μM after 48 h treatment [68]. These comparisons are constrained by variations in experimental conditions (e.g., assay method, exposure time) across studies. Notably, some findings, such as the high IC_50_ of AT-II in certain colorectal cancer cell lines, are based on single reports and warrant verification.

In animal studies, ATs significantly inhibited tumor growth and metastasis through various administration routes (e.g., intraperitoneal injection, intravenous injection, and gavage), demonstrating favorable antitumor activity (Table 2). Among xenograft mouse models, AT-I has been most extensively investigated and shows the most pronounced therapeutic effects. In BALB/c nude mice bearing T-24 bladder cancer xenografts, administration of AT-I (75 mg/kg) for 4 weeks achieved up to an 85% tumor inhibition rate [23]. Although less studied, AT-II has also shown promising potential. For instance, oral administration of AT-II (25 mg/kg) significantly reduced tumor volume and weight in nude mice bearing B16 melanoma xenografts, achieving 80% inhibition of tumor progression [49]. Generally, their in vivo antitumor efficacy increases with higher administration doses, suggesting that future studies should further optimize the dosage and delivery routes of ATs. Moreover, ATs effectively suppress tumor metastasis. In HCT15 CRC xenograft models, intraperitoneal injection of AT-II (50 mg/kg) for 30 days reduced lung metastases by 40% [57]. In contrast, studies on AT-III remain scarce. In contrast, studies on AT-III remain scarce. In nude mice bearing HCT-116 xenografts, AT-III administration significantly suppressed tumor growth [68]. This indicates the anticancer potential of AT-III, but its efficacy in different types of cancer is far less clear compared to AT-I and AT-II.

Overall, these findings suggest that AT has broad potential to inhibit tumor growth and metastasis in vivo, but they should be interpreted with caution due to inherent limitations in the existing preclinical evidence. These include the heterogeneity of animal models (e.g., varied cell lines, immunocompetent vs. immunodeficient mice), often small cohort sizes, significant variations in dosing regimens and administration routes, and a general absence of rigorous pharmacokinetic–pharmacodynamic correlations. Furthermore, most studies lack comprehensive toxicity biomarker profiles beyond body weight monitoring. These methodological inconsistencies and data gaps complicate cross-study comparisons and highlight the need for more standardized and mechanistically detailed in vivo investigations to robustly support clinical translation.

### 2.2. Combined Efficacy

Beyond their effects as monotherapy, ATs have demonstrated significant benefits in combination with anticancer therapies both in vitro and in vivo. The combination of ATs with certain drugs can enhance anticancer activity, with current research primarily focused on AT-I and AT-II. For instance, in ovarian cancer SKOV3 cells, the addition of AT-I reduced the IC50 of paclitaxel by up to 3.5-fold compared to paclitaxel monotherapy [61]. Furthermore, in lung cancer A549 and H1299 cells, co-treatment with AT-I (70 μg/mL) and the EGFR-TKI erlotinib (10 μM) for 24 h resulted in significantly greater proliferation inhibition than either agent alone [71]. Animal studies further support the anticancer efficacy of ATs in combination regimens. In MDA-MB-231 xenograft mouse models, intraperitoneal administration of AT-I (50 mg/kg) and paclitaxel (10 mg/kg) for 6 weeks inhibited tumor growth, lung metastasis, and liver metastasis by approximately 65%, 90%, and 95%, respectively, far outperforming either monotherapy [51]. Similarly, in HCT15 tumor-bearing mice, AT-II combined with IFN-γ exhibited superior efficacy in suppressing tumor growth and lung metastasis, with tumor growth inhibition rates 1.5-fold and 2-fold higher than either treatment alone [57].

The development of drug resistance in cancer cells represents a major cause of treatment failure, making the reversal of resistance a key therapeutic approach [72]. Beyond enhancing the efficacy of combination therapies, ATs demonstrate the potential to reverse drug resistance in various cancers. Specifically, AT-I was shown to overcome eIF4E-mediated resistance in prostate cancer by inhibiting the Hsp27/eIF4E pathway, thereby enhancing the chemosensitizing effects of cabozantinib [73]. Likewise, combination treatment with 5 μM sunitinib and 80 μM AT-I for 36 h abolished drug resistance in sunitinib-resistant SUR-786O cells [58]. In CRC AT-II reversed resistance to multiple chemotherapeutics (5-Fluorouracil, Mitomycin, Cisplatin, Adriamycin), reducing the IC50 of Mitomycin from 16.56 to 6.09 μg/mL in SW480 cells after 48 h treatment [56]. Furthermore, the combination of AT-II and AT-III with FDA-approved drugs enhanced sensitivity in XPC-KD-resistant renal cell carcinoma (RCC) cells, overcoming XPC deficiency-mediated resistance [74]. Animal studies have validated these anti-resistance effects, with AT-I significantly restoring paclitaxel sensitivity in triple-negative breast cancer (TNBC) xenografts and concomitantly inhibiting tumor growth/metastasis [51]. Collectively, these findings demonstrate that AT-based combination therapies can: (1) improve anticancer efficacy, (2) expand therapeutic applications, and (3) delay the emergence of resistance compared to monotherapies. However, the majority of these findings originate from single studies or specific cell line models. Robust, independent validation across diverse models is needed to confirm these synergistic interactions and establish their generalizability. Specific synergistic effects are detailed in Table 2 and Table 3.

## 3. Antitumor Mechanisms

### 3.1. Inhibition of Tumor Cell Cycle Progression

Dysregulated cell cycle progression is widely recognized as a hallmark of cancer and plays a pivotal role in tumor initiation and development, making its blockade crucial for inhibiting cancer cell proliferation [75]. ATs have been demonstrated to induce cell cycle arrest in various cancer cell lines, including bladder cancer T-24 and 253J cells [23], prostate cancer LNCaP and DU145 cells [62], lung cancer A549 cells [67], and melanoma B16 cells [76]. Of note, all three ATs (I, II, and III) can induce G1 phase arrest. For instance, treating melanoma B16 cells with 100 μM AT-I for 48 h increased the proportion of G1 phase cells from 55% to approximately 70% [76]. Additionally, AT-I and II can induce G2/M phase arrest in certain cancer models [23,62]. Specifically, 100 μM AT-II treatment for 48 h increased the proportion of prostate cancer LNCaP cells in the G2/M phase by roughly 61% compared to controls [62]. Taken together, these findings indicate that ATs induce cell cycle arrest at different phases depending on the cancer cell type, with further variations observed based on treatment duration and concentration.

The cell cycle is regulated by cyclin-dependent kinases (CDKs) [77,78], while the p21 protein inhibits CDK kinase activity by binding to cyclin-CDK complexes, thereby suppressing cell cycle progression [79]. AT-I- and AT-II-induced cell cycle arrest is typically associated with upregulation of p21 and concurrent downregulation of CDKs [23,62,65,76]. Specifically, in G2/M phase arrest, AT-II upregulates p21 expression while reducing cyclin B1 and CDK1 levels [62], potentially through inhibition of Akt activity [65] and suppression of human orthologue oncoprotein Mouse double minute 2 expression [62]. Furthermore, AT-I downregulates the expression of key cell cycle components, including cyclin A and Cdc25c [23], and additionally arrests cell cycle progression through the TLR4/MyD88 pathway [50]. In the context of G1 phase arrest, beyond mediating p21 and CDK2 expression [65,76], AT-I and AT-II may function through the ERK/GSK-3β signaling pathway. Both compounds downregulate ERK expression in melanoma B16 cells [65,76], subsequently activating GSK-3β and affecting c-Jun and cyclin expression [76]. The critical role of GSK-3β is evidenced by the partial reversal of AT-I-induced G1 arrest using LiCl (a GSK-3β inhibitor) [76]. In contrast, research on the effects of AT-III on cell cycle regulation remains limited. Current evidence from a study in lung cancer A549 cells suggests AT-III may influence the cell cycle by promoting lactate dehydrogenase release, though its precise mechanisms require further investigation [67]. Collectively, ATs induce cancer cell cycle arrest through multi-targeted modulation of cell cycle-related proteins and signaling pathways, providing an important molecular basis for antitumor therapy.

### 3.2. Regulation of Cancer Cell Programmed Cell Death

Programmed cell death is a genetically determined, active and orderly process of cellular demise, encompassing apoptosis, necroptosis, autophagy, ferroptosis, pyroptosis, and programmed necrosis [80]. By modulating these pathways, ATs can eliminate non-essential or potentially malignant cells, thereby achieving antitumor effects. Current research on ATs has largely focused on apoptosis, with limited investigations into autophagy and ferroptosis, while other forms of Programmed cell death remain largely unexplored and warrant further investigation.

#### 3.2.1. Inducing Apoptosis

ATs induce apoptosis in various cancer cells, including bladder cancer [23], breast cancer [50], CRC [52,54,55], prostate cancer [73], HCC [81] and endometrial cancer [59]. For example, treatment with 100 μM AT-II for 48 h induced apoptosis in approximately 72% of prostate cancer LNCaP cells compared to the control group [62]. Similarly, AT-III treatment significantly increased the number of apoptotic cells in HCC HepG2 and SMMC7721 cells [81]. Additionally, AT-I-treated CRC cells showed evidence of nuclear condensation, a hallmark of apoptosis [52].

ATs primarily mediate cancer cell apoptosis through the mitochondria-mediated intrinsic pathway [54,64,67]. For instance, AT-I significantly downregulates the expression of anti-apoptotic proteins (CCND1, Mcl-1, and Bcl-xl) while upregulating that of pro-apoptotic proteins (Bad, Bak, Bax, Bim, Bid, and Puma) during apoptosis induction [23,24,53,55]. This leads to mitochondrial dysfunction [54], resulting in the release of cytochrome c and Smac/Diablo into the cytosol [23]. In turn, this cascade activates caspase 3, caspase 7, caspase 9, and PARP [23,24,53,55], ultimately promoting cancer cell apoptosis. The specific signaling pathways involved vary by cancer type, including JAK2/STAT3 [49,52,62], TLR4/MyD88 [61], miR-195-5p/FGFR1 [81], PADI3-ERK [59], ERK/GSK3β [76], AKT/mTOR [55], and the Toll-Like Receptor 4-Mediated Nuclear Factor-κB Signaling Pathways [50]. Notably, excessive ROS production may serve as an additional mechanism for AT-I-induced apoptosis in CRC [54,55]. In prostate cancer, AT-I induces apoptosis in DU145 and PC-3 cells, an effect shown to be mediated, at least in part, through its direct inhibition of Hsp27 [73].

Furthermore, the combination of AT-II with IFN-γ may induce apoptosis and activate anti-tumor immunity through modulation of the Wnt/β-catenin and NF-κB p65/PD-L1 pathways [57], which likely accounts for the enhanced anti-tumor activity observed with this combination therapy [57]. The majority of AT-induced tumor cell death can be attributed to apoptosis [54]. Elucidating these mechanisms is crucial for optimizing the anti-tumor potential of ATs and developing more effective combination therapies.

#### 3.2.2. Inducing Autophagy

As is well documented, autophagy is a lysosome-dependent process for degrading cytoplasmic proteins and damaged organelles, playing a decisive role in cancer development and treatment [82,83]. ATs have demonstrated the ability to induce autophagy and enhance lysosomal degradation. For instance, in 7860 and OSRC2 RCC lines, treatment with 80 or 160 μM AT-I for 72 h promoted EPAS1 degradation via autophagy, thereby enhancing lysosomal function and accelerating autophagosome-lysosome fusion [58].

In addition, AT-I upregulates the expression of ATPase subunit ATP6V0D2 (ATPase H+ transporting V0 subunit d2) in RCC cells [58]. ATP6V0D2 directly interacts with RAB7 and VPS41, accelerating RAB7-HOPS complex formation and promoting SNARE complex assembly and autophagosome-lysosome fusion. This process enhances lysosomal acidification and activity during macroautophagy/autophagy flux, thereby facilitating autolysosomal degradation [58]. Additionally, one study suggested that AT-III may exert its effects by binding with autophagy-related proteins, such as interacting with Beclin1 at hydrophobic sites and with LC3B and ULK1 at hydrophilic sites [66]. In summary, while ATs can modulate autophagy in tumor cells, it remains unclear whether this represents their primary anticancer mechanism, given the dual role of autophagy regulation in both promoting and suppressing tumor progression across various cancers. Further investigation is required to elucidate these relationships.

#### 3.2.3. Inducing Ferroptosis

Ferroptosis is an iron-dependent form of programmed cell death characterized by the iron-dependent accumulation of lipid peroxides to lethal levels [84], which has been established as an essential mechanism for inhibiting tumor progression [85,86]. Ongoing research primarily focuses on AT-II. For instance, in studies using HCC Hep3B and Huh7 cells, treatment with specific concentrations of AT-II significantly increased the levels of Fe^2+^, lipid ROS, and malondialdehyde (MDA) compared to controls, while markedly downregulating the protein expression level of xCT and GPX4 and reducing glutathione (GSH) levels; these effects could be significantly reversed by TRAF6 overexpression [63]. As a lipid peroxide product, MDA levels are positively correlated with ferroptosis. GPX4 maintains membrane lipid bilayer homeostasis by catalytically reducing toxic lipid peroxides [87], while GSH serves as an essential cofactor for GPX4-mediated peroxide-to-alcohol conversion. GSH depletion leads to cysteine deficiency, directly inactivating GPX4 and triggering ferroptosis [88,89]. In vivo studies have reported that AT-II regulates ferroptosis in HCC by mediating the TRAF6/NF-κB pathway, ultimately reducing tumor volume and weight [63]. In conclusion, ATs induce ferroptosis in tumor cells by modulating lipid-related pathways, thereby inhibiting tumor progression both in vitro and in vivo. These findings may accelerate the development of more clinically applicable strategies and the identification of precise anticancer targets for AT-based therapies.

### 3.3. Inhibiting Tumor Angiogenesis

Tumor angiogenesis is a critical process in tumorigenesis that provides essential nutrients and oxygen to tumor tissues and is closely associated with tumor progression and metastasis [90,91]. ATs can exert antitumorigenic effects by inhibiting tumor angiogenesis. For example, in models of VHL-deficient RCC, AT-I has been shown to demonstrates significant anti-angiogenic activity, particularly in regulating vascular system development [58]. This effect is associated with its inhibition of vascular endothelial growth factor (VEGF), which promotes vascular permeability, extracellular matrix remodeling, endothelial cell migration, proliferation, and angiogenesis [92]. Mechanistically, AT-I suppresses the expression of oncogenic genes, including VEGF-A, through inhibition of the EPAS1/HIF2α pathway, effectively impeding neovascularization [58]. This mechanism may also contribute to its ability to partially reverse drug resistance in SUR-786O cells [58]. By inhibiting tumor angiogenesis, ATs not only delay tumor growth and metastasis but also provide a novel approach for overcoming tumor drug resistance.

### 3.4. Inhibiting Tumor Migration and Invasion

Tumor metastasis is a major cause of cancer-related deaths, and the migration and invasion of tumor cells are two key steps in the metastatic process. Inhibition of tumor metastasis and invasion is regarded as a core part of antitumor therapy. In addition to their antiproliferative effects, ATs have been established to inhibit the migratory and invasive abilities of tumor cells [24,50,51,55,57,60,66,73,83]. In wound healing assays using cell lines such as MDA-MB-231, MCF-7, MCF-10A, and HS578T, treatment with 50 μM AT-I/AT-III [24,50,51,66]. Specifically, the number of migrating MDA-MB-231 cells decreased from 373.14 ± 46.46 to 122.28 ± 81.78 [24]. In addition, the results of the Transwell invasion assay revealed that treatment with 50 or 100 μM AT-I significantly reduced the invasive abilities of MCF-7 and MDA-MB-231 breast cancer cells [50]. Similarly, AT-I significantly inhibited the migratory ability of A375 melanoma cells [60] and the invasive ability of CRC cells (HCT116 and COLO205) after 24 h and 72 h of treatment, respectively [55]. At the same time, one study reported that AT-III significantly reduced the migratory and invasive abilities of HCC cells (HepG2 and AMMC7721) after 24 h of treatment [81]. In vivo experiments also confirmed that the number of metastatic lung nodules in C57BL/6 mice with HCT15 lung metastases was lower in the AT-II-treated group compared to the control group [57]. It is worthwhile emphasizing that the inhibitory effects of ATs on tumor cell migration and invasion were dose-dependent [24,50,81]. The mechanisms involved may be related to the ability of ATs to inhibit epithelial–mesenchymal transition (EMT) and downregulate the level of matrix metalloproteinases (MMPs).

EMT confers migratory and invasive capabilities to cells and represents a critical determinant of cancer metastasis [93,94]. Its hallmarks include loss of epithelial markers (e.g., cytokeratins and E-cadherin) and upregulation of mesenchymal markers (e.g., N-cadherin, vimentin, and fibronectin) [95,96]. Mechanistic studies have indicated that AT-I suppresses EMT in DU145 and PC-3 prostate cancer cells, an effect associated with the silencing of Hsp27 expression, upregulating of E-cadherin, and downregulating vimentin [73]. In MCF-7 and MDA-MB-231 TNBC cells, AT-I inhibits the TLR4-mediated NF-κB signaling pathway while modulating E-cadherin and vimentin expression [50]. The HCT15 lung metastasis C57BL/6 mouse model uncovered that AT-II treatment significantly suppresses N-cadherin, Wnt, p-NF-κB p65, and PD-L1 expression while increasing E-cadherin and Smad1 levels in tumor tissues [57]. Connective tissue growth factor (CTGF), a multifunctional signaling modulator, promotes cancer initiation, progression, and metastasis through EMT regulation [97]. AT-I may reverse partial drug resistance in TNBC by downregulating CTGF expression in fibroblasts [51]. Furthermore, Nrf2 and FGFR1 mediate the EMT. Specifically, AT-III effectively inhibits E2-induced MCF-10A cell migration by promoting the autophagic degradation of Keap1 and subsequent Nrf2 downregulation [66] and suppresses cell migration through downregulation of FGFR1 and its encoded protein in HepG2 and SMMC7721 cells [81].

The migration and invasion of tumor cells are also closely associated with MMPs and related proteins [98]. Indeed, MMPs play a central role in tumor invasion and metastasis by degrading various protein components of the extracellular matrix, thereby disrupting histological barriers and driving tumor cell invasion. AT-I has been shown to reduce the protein expression levels of phosphorylated JAK2 and phosphorylated STAT3 in A375 melanoma cells, consequently downregulating the mRNA levels of STAT3 target genes MMP-2 and MMP-9 [60]. In TNBRC cells, AT-I also downregulated the expression and secretion of CTGF protein, an effect that was attenuated by CTGF-specific shRNA [51]. Similarly, treatment with 80 μmol/L AT-II alone reduced MMP-2 and MMP-9 levels in CRC HT29 and HCT15 cells [57].

### 3.5. Regulation of Tumor Immune Microenvironment

The tumor microenvironment represents a highly structured ecosystem wherein the immune microenvironment plays a vital role in cancer initiation, progression, and therapeutic response. Modulation of the tumor immune microenvironment has emerged as a crucial strategy in cancer research and treatment [99]. ATs demonstrate significant potential in mediating the tumor microenvironment.

Cellular and molecular studies have begun to delineate how ATs directly interact with immune-related pathways in tumor cells, revealing mechanisms such as checkpoint modulation and antigen presentation enhancement. For instance, AT-I may partially reverse drug resistance in epithelial ovarian cancer by inhibiting MD-2-mediated TLR4/MyD88 signaling and downregulating the expression of proinflammatory cytokines and regulatory T cells [61,100]. Additionally, Research in HCC models (e.g., Hep3B and Huh7 cells) has reported that AT-II can increase the proportion of CD8^+^ T cells and IFN-γ levels while decreasing IL-10 levels and PD-L1 expression, suggesting a role in modulating immune evasion through the TRAF6/NF-κB pathway [63]. Furthermore, molecular docking and experimental analysis in one study indicated that AT-III directly binds to the Jak3 protein (e.g., via hydrogen bonding at the Leu905-NH2 site), thereby inhibiting IFNγ-induced activation of Jak3/Stat3 pathway and its downstream target indoleamine 2,3-dioxygenase (IDO) [70]. IDO plays an immunoregulatory role in tryptophan metabolism, and inhibition of IDO activity has emerged as a promising immunotherapy strategy [101]. The mechanism involves IDO acting as a checkpoint molecule or in combination with other immune checkpoints (such as cytotoxic T-lymphocyte antigen 4 and programmed cell death protein 1) to induce T-cell suppression after tumor transformation, thereby hindering effective antitumor immune responses.

Animal model studies provide functional validation, demonstrating that ATs can remodel the tumor immune microenvironment to synergize with immunotherapies and potentiate anti-tumor immunity. For instance, when co-delivered with ginsenoside Rg1 to microsatellite-stable CRC, AT-I enhances the activity of the 26S proteasome complex in tumor cells, thereby up-regulating major histocompatibility complex class I (MHC-I) expression. This ultimately boosts cytotoxic T lymphocyte infiltration and recognition capacity in microsatellite-stable CRC, thereby elevating the tumor inhibition rate of programmed cell death protein 1 therapy from 5% to 69% [102]. Noteworthily, another study described that AT-I binding to the proteasome 26S subunit non-ATPase 4 enhances immunoproteasome-mediated antigen processing activity and MHC-I-dependent antigen presentation on cancer cells, consequently boosting CD8^+^ T cell cytotoxic responses [69].

While these in vitro and in vivo findings highlight the significant immunomodulatory potential of ATs, direct clinical evidence from human trials specifically investigating ATs as immunotherapeutic agents remains to be established. This represents a crucial avenue for future translational research.

**Figure 2:**
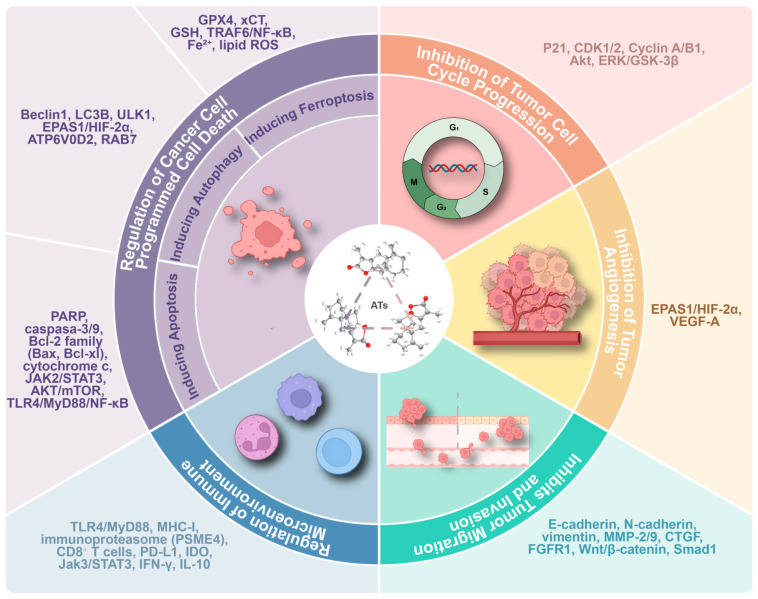
Chemical structures and the potential anticancer mechanisms of ATs.

ATs exert anticancer effects by inducing cell cycle arrest, apoptosis, autophagy, and ferroptosis, thus inhibiting tumor angiogenesis, metastasis, and regulating of tumor immune microenvironment. The representative molecular targets/pathways for key anticancer mechanisms of ATs are classified and illustrated (Figure 2). It is important to note that while some upstream targets (e.g., TLR4) have been proposed for direct interaction, many of the described signaling molecules (e.g., STAT3, NF-κB) and phenotypic outcomes represent downstream effects within the broader antitumor network.

The anticancer mechanisms of ATs, while multifaceted, are supported by varying degrees of evidence. Well-supported mechanisms, validated across multiple cancer types and independent studies, include: (1) the induction of mitochondrial apoptosis via the regulation of Bcl-2 family proteins and caspase activation [23,24,52,53,54,55]; and (2) cell cycle arrest (G1 or G2/M phase) associated with p21 upregulation and cyclin/CDK modulation [23,62,65,76]. The frequent involvement of JAK/STAT and NF-κB signaling pathways in mediating these effects also constitutes a robustly observed theme [49,52,57,60]. Mechanisms with moderate or context-dependent support include the inhibition of migration and invasion linked to EMT reversal and MMP downregulation, which, while reported in several studies, show variability depending on the cancer model [24,50,57,73]. Emerging or preliminary mechanisms, supported by compelling but currently limited evidence from specific models, encompass: (1) the induction of ferroptosis by AT-II in hepatocellular carcinoma via the TRAF6/NF-κB pathway [63]; (2) immunoproteasome activation and enhanced MHC-I antigen presentation by AT-I, primarily studied in CRC models [69,102]; (3) IDO-mediated immune modulation by AT-III via Jak3/STAT3 inhibition [70]; and (4) autophagy promotion through specific targets like EPAS1/HIF-2α or Beclin-1, which requires further validation for its precise role in antitumor outcomes [58,66]. This graded perspective clarifies the established core actions of ATs while highlighting promising, targetable avenues for future validation.

## 4. Pharmacokinetics

Mounting evidence suggests that the anticancer efficacy of ATs generally increases with higher doses and prolonged treatment duration, necessitating enhanced pharmacokinetic studies for more efficient and precise dosing. Currently administered primarily via the oral route, ATs are absorbed through the intestinal tract into the systemic circulation and exhibit tissue-specific distribution patterns. AT-I and AT-II predominantly accumulate in the spleen, liver, and kidneys, with detectable levels in the heart and brain [103], while AT-III shows higher pulmonary distribution, with higher levels in the liver, spleen, kidneys, and pancreas compared to the heart [104], while maintaining blood–brain barrier permeability [105]. Notably, cardiac concentrations remain the lowest for all ATs [103,104], yet AT-II is exclusively detectable in the heart 12 h post-administration [103]. Metabolically, ATs exhibit both saponin-like characteristics and water solubility [106], undergoing metabolism primarily through hepatic biotransformation enzymes and splenic [107] and renal [108] excretion mechanisms [109]. The structurally similar AT-I and AT-II share parallel metabolic pathways, including sulfation, oxidation, and desaturation [103], with AT-II capable of undergoing oxidative conversion to AT-I [110]. In contrast, AT-III undergoes a more complex metabolism involving hydroxylation, epoxidation, hydration, N-acetylcysteine conjugation, and glucuronidation [111,112]. Ultimately, the metabolites can be detected in rat feces, urine, and plasma.

Quantitative analysis of ATs’ metabolic kinetics in rat plasma using LC-MS/MS has demonstrated satisfactory linearity for AT-I, AT-II, and AT-III within certain concentration ranges [31,113]. Key pharmacokinetic parameters, including AUC_(0-t)_ (area under the plasma concentration-time curve from 0 to infinity), MRT (mean residence time), t_1/2_ (biological half-life), T_max_ (time to peak concentration), and C_max_ (peak plasma concentration), are detailed in Table 4. The pharmacokinetic properties of ATs are influenced by various factors such as processing methods and co-administration. Compared to raw *A. macrocephala*, bran processing significantly increases the plasma levels of AT-I, AT-II, and AT-III, with marked elevations in C_max_ and AUC_(0-t)_ values [114]. Furthermore, the traditional Chinese formula Sijunzi decoction increases AT-III levels in tissues [104], while biomimetic iron-porphyrin complexes can catalyze the in vivo metabolism of atractylenolide III [115]. Interestingly, oral administration studies in rats have unveiled significant gender differences in AT-III pharmacokinetics [48,116]. Female rats showed higher plasma concentrations of AT-III compared to males, with detectable levels persisting for 12 h post-administration compared to only 2 h in males. Other pharmacokinetic parameters, including C_max_, AUC_(0-t)_, and T_1/2_, were also significantly higher in females [48]. Research suggests intestinal absorption and hepatic metabolism as key factors underlying this sexual dimorphism—female rat liver microsomes contained 30% more AT-III than males, indicating slower metabolic rates in females [117].

Earlier studies have partially elucidated the pharmacokinetic patterns of ATs (Table 4), yet these findings remain insufficient. A critical translational gap persists: the systemic exposures (PK parameters) reported in these studies are rarely directly correlated with the drug concentrations required to achieve the antitumor efficacy observed in in vivo models. Bridging this pharmacodynamic gap is essential for reliable human dose projection. The clinical translation of ATs is confronted by several interconnected challenges rooted in their physicochemical and metabolic properties: (1) Bioavailability and Absorption: As sesquiterpene lactones, their oral bioavailability may be limited by solubility and first-pass metabolism; (2) Complex Disposition and Variability: The considerable inter-study variability in key pharmacokinetic parameters—attributable to factors like differing herbal extraction methods, use in complex formulae, and animal models—coupled with observed gender-dependent metabolism (e.g., for AT-III), suggests a potential for unpredictable exposure in humans; (3) Tissue Distribution and Targeting: While distribution to organs like the liver and spleen is documented, achieving sufficient and selective intratumoral concentrations remains a key hurdle. (4) Safety and Interaction Potential: As components of traditional formulae, ATs’ metabolism potentially involving cytochrome P450 enzymes raises concerns about drug–herb interactions, especially when combined with conventional chemotherapeutics, necessitating rigorous interaction studies. To advance ATs towards clinical application, future research must prioritize pharmacokinetics-pharmacodynamics modeling in tumor-bearing animals at therapeutic doses to define effective exposure ranges. Concurrently, developing advanced delivery systems (e.g., nanoparticle encapsulation, lipid-based formulations) is a promising strategy to simultaneously address the challenges of solubility, bioavailability, targeted delivery, and potentially, mitigated toxicity.

The anticancer profiles of the three natural products Atractylenolide I, II, and III have both similarities and differences, which are compared in Table 5. This table outlines the differences and overlapping features of Atractylenolide I, II, and III, facilitating comparative analysis and proposing hypotheses for future research.

## 5. Safety

Current evidence regarding the safety profile of ATs is primarily derived from preclinical animal models and a limited number of pilot clinical investigations. These studies have consistently reported no significant changes in body weight or overt signs of toxicity at doses demonstrating antitumor efficacy, suggesting a potentially favorable therapeutic window in the contexts examined. In animal studies, tumor-bearing mice receiving 50 mg/kg AT-I or AT-II via gavage exhibited lower tumor volume and weight without significant body weight changes compared to controls [52,54,63], indicating low toxicity. Rats administered 1.2 mg/kg/day AT-III for 10 weeks exhibited no evidence of toxicity or behavioral alterations [119]. More importantly, mice tolerated intraperitoneal injection of up to 75 mg/kg AT-I for 4 weeks without adverse effects [23]. A randomized (but not blinded) pilot study in cachexic gastric cancer patients demonstrated no signs of toxicity following twice-daily 0.66 mg AT-I administration for 7 weeks [120].

However, a comprehensive and definitive safety assessment is constrained by several critical limitations inherent in the available data: (1) Limited Scope and Duration of Exposure: Most toxicological observations are from studies with treatment periods spanning only a few weeks. This short-term exposure may fail to reveal chronic, cumulative, or delayed toxicities that could arise from prolonged administration. (2) Insufficient Depth of Organ Toxicity Evaluation: Assessments have predominantly relied on gross measures such as body weight and clinical observation. There is a general lack of detailed histopathological analyses of vital organs (e.g., liver, kidneys, heart, lungs) and specific biochemical or functional biomarkers for organ dysfunction following either acute high-dose or chronic administration. (3) Scarcity of Data on Combination Therapy Safety: A significant knowledge gap exists concerning the potential of ATs to modulate cytochrome P450 enzymes and other drug-metabolizing pathways. This unexplored area raises valid concerns about possible herb-drug interactions. There is a lack of systematic and long-term studies on its safety profile when used in combination with other drugs, especially standard anticancer medications, including chemotherapy and immunotherapy. (4) Uncertainty Regarding Class-Specific Risks: As sesquiterpene lactones, ATs carry a theoretical risk for compound-specific adverse effects, such as idiosyncratic reactions or hepatotoxicity, which warrants proactive monitoring in future preclinical and clinical studies.

Therefore, while the preliminary data are encouraging, claims of definitive safety or “non-toxicity” are premature. A robust safety profile that can reliably inform clinical development must be established through dedicated, rigorous toxicological studies. Future investigations must prioritize studies with extended duration, incorporate comprehensive organ histopathology and biomarker analyses, systematically evaluate safety in combination with standard therapies, and characterize metabolic interaction potential. Addressing these gaps is essential to thoroughly de-risk the clinical translation of ATs.

## 6. Discussion and Future Perspectives

This review has synthesized the current landscape of research on the anticancer potential of atractylenolides I, II, and III. The compiled data substantiate that these sesquiterpene lactones, particularly AT-I and AT-II, are promising natural product-derived agents with broad-spectrum activity across diverse in vitro and in vivo cancer models [23,24,25,26,49,52,57]. Their actions are pleiotropic, engaging multiple hallmarks of cancer through mechanisms ranging from cell cycle arrest and apoptosis to the modulation of ferroptosis and the tumor immune microenvironment [57,58,63,69]. However, a critical and integrative analysis reveals significant gaps between these promising preclinical findings and tangible clinical translation. This section aims to move beyond descriptive summarization, offering a comparative synthesis of the evidence, a candid appraisal of existing limitations, and a prioritized roadmap for future research.

### 6.1. Differentiated Profiles: A Comparative Analysis of AT-I, AT-II, and AT-III

A critical synthesis reveals distinct profiles for each compound. AT-I possesses the most extensive dataset, demonstrating potent efficacy in bladder, breast, and colorectal cancers, with strong evidence for mechanisms involving JAK2/STAT3 inhibition and immunoproteasome-mediated antigen presentation [23,24,52,69]. AT-II shows pronounced activity in melanoma, prostate cancer, and HCC, with a unique and well-documented role in inducing ferroptosis via the TRAF6/NF-κB pathway [49,62,63]. In contrast, AT-III exhibits generally higher IC_50_ values in vitro but demonstrates unique mechanisms, such as Nrf2/Keap1 regulation and IDO/Jak3-STAT3 inhibition, suggesting a potential niche in chemoprevention or combination immunotherapy [66,70]. This disparity highlights that the “ATs” cannot be considered a monolithic entity; their therapeutic applications may be context and compound-specific.

### 6.2. Critical Appraisal: Four Key Barriers on the Path to Translation

Despite the promise, the translation of ATs is hindered by several interconnected barriers that must be explicitly acknowledged.

#### 6.2.1. Heterogeneity and Lack of Standardization in Preclinical Evidence

In vivo studies employ vastly different animal models, dosing regimens, and efficacy endpoints, making direct comparison and reliable meta-analysis challenging [23,49,52,57]. Many studies also lack robust pharmacodynamic correlates and detailed toxicity biomarker analysis, limiting the strength of conclusions.

#### 6.2.2. The Pharmacokinetic–Pharmacodynamic Disconnect

While pharmacokinetic studies in healthy rodents outline absorption and distribution patterns, a critical gap exists in correlating these parameters with the actual exposures achieved at efficacious doses in tumor-bearing models [103,104,114]. Fundamental challenges inherent to sesquiterpene lactones, such as oral bioavailability and tissue-specific targeting, remain understudied for ATs [109].

#### 6.2.3. Insufficient Mechanistic Depth and Target Specificity

Although numerous pathways are implicated, the direct molecular targets for most ATs remain unidentified. The extrapolation of mechanisms from single cell-line studies is common, and systematic structure–activity relationship studies are absent, hindering rational drug design and optimization [121].

#### 6.2.4. An Incomplete and Fragmented Safety Profile

Current claims of low toxicity are primarily based on the absence of gross weight loss or overt symptoms in short-term studies [23,52,120]. Data are critically lacking on long-term organ histopathology, potential cytochrome P450-mediated drug–herb interactions, and safety in combination with standard chemo- or immunotherapies, precluding a definitive safety designation.

### 6.3. A Roadmap for Future Research: Priority Actions

To advance ATs from promising leads to clinical candidates, focused efforts should address the following priorities:

#### 6.3.1. Conduct Head-to-Head Comparative Studies

Direct in vivo comparisons of AT-I, AT-II, and AT-III in standardized, immunocompetent models are needed to definitively rank their efficacy, toxicity, and mechanism-of-action profiles against specific cancer types. In addition, future studies should include direct comparisons of ATs with relevant standard chemotherapeutics in standardized assays to better contextualize their potency and potential therapeutic window.

#### 6.3.2. Overcome Pharmaceutical and Pharmacokinetic–Pharmacodynamic Hurdles

Research must prioritize strategies to improve druggability, such as developing novel formulations (nanoparticles, liposomes) to enhance solubility and bioavailability [115]. Integrated pharmacokinetic–pharmacodynamic studies in relevant disease models are essential to define therapeutic windows.

#### 6.3.3. Elucidate Direct Targets and Enable Rational Design

Employing chemical proteomics and other target-deconvolution strategies is crucial to identify the direct protein interactors of ATs. This knowledge must then drive structure–activity relationship studies to design more potent and selective analogs [122].

#### 6.3.4. Develop Mechanism-Informed Combination Therapies

Preclinical studies should systematically evaluate rational combinations, such as AT-I with PD-1 inhibitors for CRC or AT-II with standard-of-care therapies in HCC, leveraging their distinct immunological and ferroptosis-inducing properties [57,100].

#### 6.3.5. Design Biomarker-Driven Early-Phase Clinical Trials

For the most compelling candidates, pilot clinical trial concepts should be developed. These trials should incorporate biomarker hypotheses (e.g., PD-L1 status, ferroptosis-related gene signatures) to enrich patient populations and provide proof-of-concept for clinical activity.

### 6.4. Concluding Remarks

Atractylenolides I–III represent a valuable scaffold for anticancer drug discovery, offering multi-target mechanisms and favorable preliminary safety. However, their development is currently at a preclinical crossroads. By addressing the identified limitations through rigorous, comparative, and translational science, the potential of these natural compounds can be effectively evaluated and harnessed. Future work must move beyond descriptive reporting to hypothesis-driven research that clarifies their unique advantages and integrates them into the modern oncology therapeutic arsenal.

## Figures and Tables

**Table 1:** Anti-proliferative activities of AT-I, AT-II and AT-III in vitro (Treatment Alone).

Types	Cancer Type	Cell Line	Time (h)	Method	Lowest IC_50_	Reference
AT-I	Breast cancer	MDA-MB-231	24	CCK-8	33.79 μM	[24]
		MDA-MB-231	48	CCK-8	32.64 μM	
		MDA-MB-231	72	CCK-8	33.91 μM	
	Breast cancer	MDA-MB-231	24	MTT	164 μM	[50]
		MDA-MB-231	48	MTT	139 μM	
		MDA-MB-231	72	MTT	105 μM	
		MCF-7	24	MTT	251 μM	
		MCF-7	48	MTT	212 μM	
		MCF-7	72	MTT	172 μM	
	Colorectal cancer	HCT116	24	CCK-8	126.8 μM	[52]
		HCT116	48	CCK-8	98.49 μM	
		SW480	24	CCK-8	97.19 μM	
		SW480	48	CCK-8	70.44 μM	
	Colorectal cancer	HT-29	24	MTT	277.6 μM	[53]
		HT-29	48	MTT	95.7 μM	
		HT-29	72	MTT	57.4 μM	
	Colorectal cancer	HCT116	24	CCK-8	2736 μM	[54]
	Colorectal cancer	COLO205	72	MTT	150–200 μM	[55]
	Bladder cancer	T-24	48	MTT	12.8 μM	[23]
		253J	48	MTT	63.7 μM	
		RT4	48	MTT	44.5 μM	
		5637	48	MTT	18.4 μM	
	Kidney cancer	HK2	48	CCK-8	657.4 μM	[58]
		ACHN	48	CCK-8	285.7 μM	
		OSRC2	48	CCK-8	125 μM	
		786O	48	CCK-8	75.77 μM	
	Endometrial cancer	RL95-2	24	CCK-8	about 100 μM	[59]
		RL95-2	48	CCK-8	100–200 μM	
		RL95-2	72	CCK-8	about 200 μM	
	Melanoma	A375	24	MTT	about 150 μM	[60]
		A375	48	MTT	about 100 μM	
		A375	72	MTT	about 100 μM	
	Lung cancer	A549	48	MTT	20–40 μM	[26]
	Leukemia	HL-60	12	MTT	46 μM	[25]
AT-II	Colorectal cancer	HT29	24	CCK-8	1727 μM	[57]
		HT29	48	CCK-8	272.5 μM	
		HCT15	24	CCK-8	490.6 μM	
		HCT15	48	CCK-8	1.58 μM	
	Prostate cancer	DU145	48	MTT	94 μM	[62]
		DU145	72	MTT	47 μM	
		LNCaP	48	MTT	100 μM	
		LNCaP	72	MTT	49 μM	
	Hepatocellular carcinoma	Hep3B	48	CCK-8	96.43 μM	[63]
		Huh7	48	CCK-8	118.38 μM	
	Melanoma	B16	48	MTT	82.3 μM	[65]
	Gastric cancer	HGC-27	72	MTT	100–200 μM	[64]
AT-III	Breast cancer	MCF-10A	24	CCK-8	>1600 μM	[66]
		MCF-10A	48	CCK-8	>1600 μM	
		MCF-10A	72	CCK-8	>1600 μM	
	Colorectal cancer	HCT116	48	CCK-8	111.47 μM	[68]
	Lung cancer	A549	24	MTT	>100 μM	[67]

Note: For consistency and ease of comparison, all IC_50_ values in Table 1 are presented in µM. Conversions were performed using the respective molecular weights of the compounds.

**Table 2:** In vivo anticancer activities of AT-I, AT-II, and AT-III.

Types	Cancer Type	Animal Model	Cell Line	Mode of Administration	Highest Tumor Growth Inhibition Rate	Reference
AT-I	Colorectal cancer	Balb/c-nu/nu nude mice	HCT116	75 mg/kg/day for 22 days, i.p ^1^	approximately 30%	[55]
	Colorectal cancer	BALB/c nude mice	HCT116	50 mg/kg/day for 3 weeks, i.p	approximately 65%	[52]
	Colorectal cancer	C57BL/6J mice	AOM/DSS-induced CRC	50 mg/kg/bid for 10 weeks, i.g ^2^	approximately 30%	[54]
	Colorectal cancer	C57BL/6 mice	MC38	50 mg/kg/day for 22 days, i.p	approximately 20%	[69]
	Breast cancer	BALB/c nude mice	MDA-MB-231	25, 50 mg/kg every 2 days for 24 days, i.v ^3^	approximately 40%, 50%	[24]
	Breast cancer	BALB/c nude mice	MDA-MB-231	AT-I 50 mg/kg/day for 6 weeks, i.p	approximately 5% Liver metastasis inhibition rate was about 5% Lung metastasis inhibition rate was about 5%	[51]
				Paclitaxel 10 mg/kg/day for 6 weeks, i.p	approximately 35% Liver metastasis inhibition rate was about 60% Lung metastasis inhibition rate was about 65%	
				AT-I 50 mg/kg/day + Paclitaxel 10 mg/kg/day for 6 weeks, i.p	approximately 65% Liver metastasis inhibition rate was about 90% Lung metastasis inhibition rate was over 95%	
	Bladder cancer	BALB/c nude mice	T-24	50, 75 mg/kg/day for 4 weeks, i.p	approximately 75%, 85%	[23]
		BALB/c nude mice	253J	50, 75 mg/kg/day for 4 weeks, i.p	approximately 40%, 80%	
	Lung cancer	BALB/c nude mice	A549	40 mg/kg/day for 16 days, i.p	approximately 50%	[26]
AT-II	Colorectal cancer	BALB/c nude mice	HCT15	AT-II 50 mg/kg/day for 3 weeks, i.p	approximately 55%	[57]
				IFN-γ 0.3 mg/kg/day for 3 weeks, i.p	approximately 35%	
				AT-II 50 mg/kg/day + IFN-γ 0.3 mg/kg/day for 3 weeks, i.p	approximately 75%	
		C57BL/6 mice	HCT15	50 mg/kg/day for 30 days, i.p	Lung metastasis inhibition rate was about 40%	
	Melanoma	C57/BL6 mice	B16	25 mg/kg/day for 14 days, i.g	approximately 80%	[49]
	Liver cancer	BALB/c nude mice	Huh7	50 mg/kg/day for 4 weeks, i.g	approximately 50%	[63]
AT-III	Colorectal cancer	BALB/c-nu mice	HCT116	200 mg/kg/day for 30 days, i.g	approximately 64%	[68]
	Lung cancer	C57BU6 mice	LCC	100 mg/kg/day for 3 weeks, i.g	approximately 30%	[70]

^1^ intraperitoneal injection, ^2^ gavage, ^3^ intravenous injection.

**Table 3:** Anti-proliferative activities of AT-I and AT-II in vitro (Combination Treatment).

Drugs	Cancer Type	Cell Line	Time (h)	Method	Combined Medication	Lowest IC_50_ (Drugs)	Reference
Paclitaxel	Breast cancer	MDA-MB-231	48	CCK-8	-	-	[51]
					AT-I	Reduced by about a factor of 1	
		HS578T	48	CCK-8	-	-	
					AT-I	Reduced by about a factor of 1	
	Ovarian cancer	SKOV3	72	MTT	-	0.038 μM	[61]
					AT-II	0.011 μM	
5-Fluorouraci	Colorectal cancer	SW480	48	MTT	-	96.18 μM	[56]
					AT-II	78.72 μM	
		Lovo	48	MTT	-	84.56 μM	
					AT-II	77.42 μM	
Mitomycin	Colorectal cancer	SW480	48	MTT	-	49.53 μM	
					AT-II	18.22 μM	
		Lovo	48	MTT	-	71.48 μM	
					AT-II	29.91 μM	
Cisplatin	Colorectal cancer	SW480	48	MTT	-	38.63 μM	
					AT-II	30.53 μM	
		Lovo	48	MTT	-	56.43 μM	
					AT-II	49.83 μM	
Adriamycin	Colorectal cancer	SW480	48	MTT	-	13.75 μM	
					AT-II	8.19 μM	
		Lovo	48	MTT	-	24.16 μM	
					AT-II	11.12 μM	

Note: For consistency and ease of comparison, all IC_50_ values in Table 3 are presented in µM. Conversions were performed using the respective molecular weights of the compounds.

**Table 4:** Pharmacokinetic parameters of AT-I, AT-II and AT-III.

Types	Animal	Method	Dose	Administration Route	AUC_(0-t)_ (ng/mL h)	MRT (h)	T_1/2_ (h)	T_max_ (h)	C_max_ (ng/mL)	Reference
AT-I	Rat (male)	UPLS-MS/MS	3.75 g/kg raw Atractylodis Rhizoma (equal to 0.48 mg/kg of AT-I)	p.o ^1^	116.75 ± 18.38	5.28 ± 1.36	3.58 ± 1.69	1.5 ± 0	32.09 ± 2.05	[114]
			3.75 g/kg wheat Bran-processed Atractylodis Rhizoma extract solution (equal to 0.46 mg/kg of AT-I)	p.o	219.14 ± 46.65	4.86 ± 0.93	2.29 ± 1.18	1.5 ± 0	66.94 ± 10.89	
	Rat (male+female)	LC-MS/MS	20 g/kg Atractylodis extract (equal to 10.6 mg/kg of AT-I)	p.o	22.2 ± 1.9	-	1.94 ± 0.27	0.81 ± 0.11	7.99 ± 1.2	[117]
AT-II	Rat (male)	UPLS-MS/MS	3.75 g/kg raw Atractylodis Rhizoma (equal to 0.6 mg/kg of AT-II)	p.o	181.21 ± 29.35	5.93 ± 3.14	4.12 ± 4.12	1.5 ± 0	49.62 ± 7.69	[114]
			3.75 g/kg wheat Bran-processed Atractylodis Rhizoma extract solution (equal to 0.75 mg/kg of AT-II)	p.o	202.43 ± 68.52	5.03 ± 1.58	4.02 ± 3.10	1 ± 0	55.9 ± 13.58	
	Rat (male)	LC-MS/MS	1.2 g/kg Atractylodis extract (equal to 82.81 mg/kg of AT-II)	p.o	28.46 ± 7.71	-	2.63 ± 1.08	0.67 ± 0.39	7.99 ± 0.90	[118]
	Rat (female)	UPLS-MS/MS	4 g/kg XYP ^2^ extract (equal to 4.04 mg/kg of AT-II)	p.o	69.53 ± 25.72	4.43 ± 0.98	3.54 ± 1.21	0.64 ± 0.29	22.48 ± 8.04	[47]
AT-III	Rat (male)	UPLS-MS/MS	3.75 g/kg raw Atractylodis Rhizoma (equal to 0.68 mg/kg of AT-III)	p.o	230.62 ± 76.76	3.48 ± 0.29	1.56 ± 0.61	1 ± 0	87.04 ± 17.03	[114]
			3.75 g/kg wheat Bran-processed Atractylodis Rhizoma extract solution (equal to 0.56 mg/kg of AT-III)	p.o	284.83 ± 32.94	3.07 ± 0.31	1.81 ± 0.79	1 ± 0	11,310 ± 19.04	
	Rat (male)	UPLS-MS/MS	6 g/kg YCZFD ^3^ (equal to 1.218 mg/kg of AT-III)	p.o	37.56 ± 14.62	2.04 ± 0.96	3.04 ± 1.43	0.33 ± 0.14	16.07 ± 3.32	[48]
	Rat (male)	LC-MS/MS	2 g/mL C. pilosula alcoholic extract (equal to 12 mg/kg of AT-III)	p.o	333.88 ± 55.3	14.92 ± 1.38	15.85 ± 2.73	0.083	21.97 ± 3.13	[116]
	Rat (female)	UPLS-MS/MS	6 g/kg YCZFD (equal to 1.218 mg/kg of AT-III)	p.o	124.77 ± 18.38	4.07 ± 0.03	7.67 ± 0.78	0.50 ± 0.00	34.37 ± 2.98	[48]
	Rat (female)	LC-MS/MS	2 g/mL C. pilosula alcoholic extract (equal to 12 mg/kg of AT-III)	p.o	2379.59 ± 803.32	13.25 ± 0.98	12.64 ± 1.19	1.0	320 ± 147.83	[116]
	Rat (female)	UPLS-MS/MS	4 g/kg XYP extract(equal to 5.68 mg/kg of AT-III)	p.o	1044.70 ± 496.68	8.60 ± 3.13	6.95 ± 2.65	0.67 ± 0.26	299.66 ± 107.94	[47]

^1^ peros. ^2^ Xiaoyao Powder, a classic TCM formula, is composed of Radix Bupleuri, Radix Angelicae Sinensis, Radix Paeoniae Alba, Rhizoma Atractylodis Macrocephalae, Poria, Radix Glycyrrhizae, Herba Menthae, and Rhizoma Zingiberis Recens. ^3^ Yinchenzhufu decoction, a classic TCM formula, is composed of Artemisiae scopariae herba, Atractylodis macrocephalae Rhizoma, Glycyrrhizae Radix et Rhizoma Praeparata cum melle, Aconiti lateralis Radix Praeparata, Zingiberis Rhizoma, and Cinnamomi cortex.

**Table 5:** Comparative overview of the anticancer profiles of Atractylenolide I, II, and III.

Types	Main Tumor Types Studied (Selected)	Key Anticancer Mechanisms (with Best Evidence)	Representative In Vivo Efficacy	Notable Immune-Microenvironment Effects
AT-I	Bladder cancer (T-24, 5637) [23]; Triple-negative breast cancer (MDA-MB-231) [24,51]; Leukemia (HL-60) [25]; Lung cancer (A549) [26]; Renal cell carcinoma (786O) [58].	· Apoptosis via JAK2/STAT3 inhibition [52] and mitochondrial pathway [23,54]. · Cell cycle arrest (G1/G2-M phase) [23,76]. · Emerging: Immunoproteasome activation and MHC-I ^3^ presentation [69,102]; Anti-angiogenesis via EPAS1/HIF2α [58].	Model: BALB/c nude mice with T-24 xenografts [23]. Result: ~85% tumor inhibition at 75 mg/kg (i.p ^1^).	Enhances cytotoxic T lymphocyte infiltration in CRC by boosting MHC-I antigen presentation [69,102].
AT-II	Melanoma (B16) [49,65]; Prostate cancer (LNCaP, DU145) [62]; Colorectal cancer (HCT115) [57].	· Induction of ferroptosis via TRAF6/NF-κB pathway in HCC [63]. · Apoptosis and cell cycle arrest via JAK2/STAT3 and Akt/GSK-3β pathways [62,65]. · Emerging: Synergy with IFN-γ via Wnt/β-catenin and PD-L1 modulation [57].	Model: C57/BL6 mice with B16 xenografts [49]. Result: ~80% tumor inhibition at 25 mg/kg (i.g ^2^).	Modulates tumor immune contexture; increases CD8+ T cells, decreases PD-L1 in HCC models [63].
AT-III	Colorectal cancer (HCT116) [68]; Hepatocellular carcinoma (HepG2) [81]	· Inducing apoptosis in CRC through the Bax/Bcl-2 pathway [68]. · Emerging/Unique: Activation of Nrf2/Keap1-autophagy axis for chemoprevention [66]. · Emerging/Unique: Inhibition of IDO ^4^-mediated immunosuppression via Jak3/STAT3 pathway [70]. · Apoptosis via mitochondrial pathway in lung cancer [67].	Model: BALB/c-nu mice with CRC xenografts [68]. Result: ~64% tumor inhibition at 200 mg/kg (i.g).	Suppresses IDO activity, potentially reversing tumor-induced T-cell suppression and synergizing with immunotherapy [70].

^1^ intraperitoneal injection, ^2^ gavage, ^3^ major histocompatibility complex class I, ^4^ indoleamine 2,3-dioxygenase.

## Data Availability

Where no new data were created.

## Abbreviations

The following abbreviations are used in this manuscript:

ATs	Atractylenolides
AUC_(0-t)_	area under the plasma concentration-time curve from 0 to infinity
CDKs	cyclin-dependent kinases
C_max_	peak plasma concentration
CRC	colorectal cancer
CTGF	Connective tissue growth factor
EMT	epithelial–mesenchymal transition
GSH	glutathione
HCC	hepatocellular carcinoma
IDO	indoleamine 2,3-dioxygenase
MDA	malondialdehyde
MHC-I	major histocompatibility complex class I
MMPs	matrix metalloproteinases
MRT	mean residence time
RCC	renal cell carcinoma
t_1/2_	biological half-life
T_max_	time to peak concentration
TNBC	triple-negative breast cancer
TCM	Traditional Chinese Medicine
VEGF	vascular endothelial growth factor
